# MSClique: Multiple Structure Discovery through the Maximum Weighted Clique Problem

**DOI:** 10.1371/journal.pone.0145846

**Published:** 2016-01-14

**Authors:** Gerard Sanroma, Adrian Penate-Sanchez, René Alquézar, Francesc Serratosa, Francesc Moreno-Noguer, Juan Andrade-Cetto, Miguel Ángel González Ballester

**Affiliations:** 1 Univ. Pompeu Fabra, Dept. of Information and Communication Technologies, Tánger 122-140, 08018 Barcelona, Spain; 2 University College London, Dept. of Computer Science, Gower Street, London WC1E 6BT, United Kingdom; 3 Univ. Politècnica de Catalunya, Institut de Robòtica i Informàtica Industrial, Llorens Artigas 4-6, 08028 Barcelona, Spain; 4 Univ. Rovira i Virgili, Dept. d’Enginyeria Informàtica i Matemàtiques, Av. Països Catalans 26 Campus Sescelades, 43007 Tarragona, Spain; 5 ICREA, Pg. Lluis Companys 23, 08010 Barcelona, Spain; University of North Carolina, UNITED STATES

## Abstract

We present a novel approach for feature correspondence and multiple structure discovery in computer vision. In contrast to existing methods, we exploit the fact that point-sets on the same structure usually lie close to each other, thus forming clusters in the image. Given a pair of input images, we initially extract points of interest and extract hierarchical representations by agglomerative clustering. We use the maximum weighted clique problem to find the set of corresponding clusters with maximum number of inliers representing the multiple structures at the correct scales. Our method is parameter-free and only needs two sets of points along with their tentative correspondences, thus being extremely easy to use. We demonstrate the effectiveness of our method in multiple-structure fitting experiments in both publicly available and in-house datasets. As shown in the experiments, our approach finds a higher number of structures containing fewer outliers compared to state-of-the-art methods.

## Introduction

Robust discovery of multiple and corresponding objects in images is a fundamental problem with a wide range of applications in computer vision, such as structure from motion, robot localization and tracking. The solution to this problem usually starts with a set of putative correspondences obtained based on the similarity of the keypoints extracted from two images. Eventual recognition of objects can be accomplished by exploiting the prior knowledge about the geometric transformations undergone by the object [1–7]. For example, we know that any two corresponding point-sets lying on planar objects are related by a homography transformation [8]. This is the main intuition behind RANSAC [9], which iteratively computes the mapping between two point sets while estimating the correspondences. Generalization of RANSAC-based methods to the case of multiple objects is of great practical interest for the computer vision community. However, correct segmentation of the multiple structures from noisy inputs is an elusive problem due to the difficulties of handling more than one transformation simultaneously. Such great practical interest has motivated the emergence of novel, more elaborate, methods at the intersection of robust estimation, segmentation and clustering.

For example, Chin *et al*. present Multi-GS [10], a method that generates multiple structure hypotheses using guided sampling, where correspondences belonging to the same structure are more likely to be sampled together. Cho *et al*. proposes agglomerative correspondence clustering (ACC) [11], which poses the multiple structure discovery as a clustering problem, where structures are regarded as clusters of correspondences.

In this paper, we propose a different perspective to discover multiple structures given point-sets in two images. Contrarily to previous methods, we use the fact that structures are most likely found in contiguous regions in space and thus, points belonging to the same structure can be grouped into one (or a few) clusters. For this purpose, we adopt a divide-and-conquer strategy in which we initially divide the two point-sets into hierarchies of clusters through agglomerative clustering and then estimate the cluster-wise correspondences, representing the structures at the correct scales, through the Maximum Weighted Clique Problem (MWCP).

**Fig 1:**
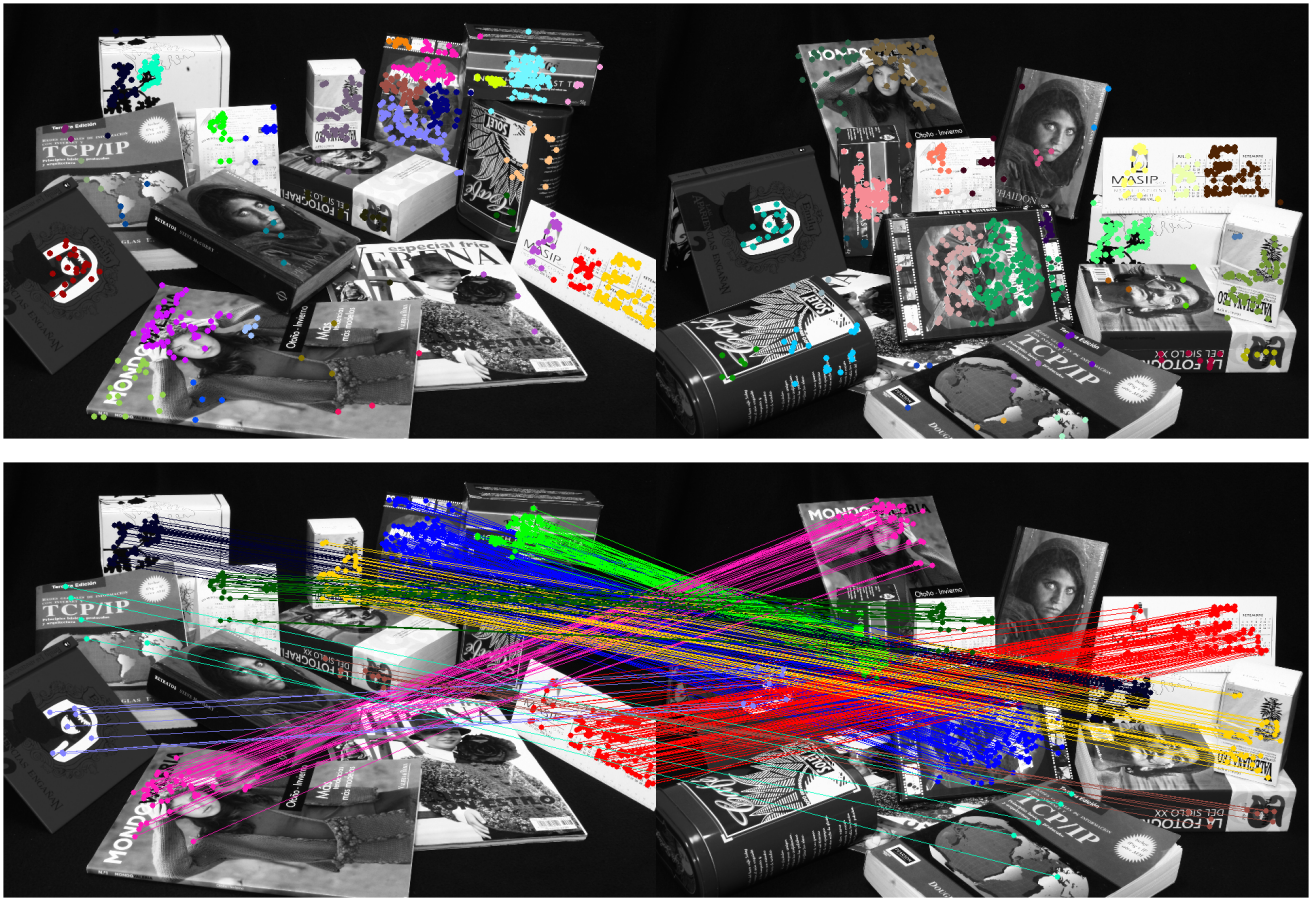
Example of the multiple structures between two images we discover using our method. We use a coarse-to-fine approach. The top image shows the initial clusters at the lower-level of the agglomerative clustering process. The bottom image depicts the final corresponding structures found by our method. Note that we are able to match structures even under large perspective and scale changes.

In the experimental section, we show that our method finds either more, or a similar, number of structures than competing methods, but consistently containing smaller amounts of incorrect matches. Additional advantages with respect to state of the art, is that we can even match the smallest structures between images and we do not merge objects which are far apart (see Fig 1). Our method is also robust to spurious structures since outlier correspondences will unlikely form bundles between two clusters. Furthermore, we do not require to tune any parameter other than the internal RANSAC threshold to obtain the consistent matches.

## Related Work

Relatively few approaches have been reported to solve the problem of multi-structure geometric model fitting. Some of them simply apply RANSAC [9] sequentially, i.e. fit one structure, remove the corresponding inliers, and iterate to find subsequent structures [12]. This approach has two main problems: (1) inaccuracies in the initial fit are amplified in subsequent fits [13], and (2) finding a stopping criterion to reach a correct number of structures is not easy.

Some methods generate a large number of hypotheses and then, assuming that the optimal ones are among the initial set, find the subset that maximize the fitting likelihood. For example, Lazic *et al*. [14] pose the multiple-structure fitting problem as a facility location problem. Given a set of points in the images, they first randomly generate a large set of candidate models, afterwards, the problem is solved by finding the optimal point-to-structure assignment that minimizes both the sum of distances from points to their assigned structures and the sum of complexities of the selected structures. They ensure that the selected structures are representative of the data and prevent overly complex solutions with too many structures. Isack and Boykov [15] also propose to find multiple structures by solving a similar energy minimization problem initialized using a set of candidate structures. However, as opposed to [14], the structures are iteratively refined using the assignment results at each iteration. Another novelty of this method, is that points that are close in space are encouraged to belong to the same structure. The regularization models used by the previous methods, use a criterion that penalizes the complexity of the solution. In [16], a novel regularization approach was introduced that penalizes the overlap between pairs of structures to find the existing structures in the image.

Instead of using a pre-defined set of candidate structures, other methods sample the hypotheses on-the-fly during the optimization procedure using a guided matching approach. The ITKSF method presented in [17] proposes a hypothesis sampling scheme based on incremental construction of distances between partial rankings (the so-called *top-k lists*) derived from residual sorting information. Wong et *al*. [18] present a similar approach that uses mode seeking in the space of permutations defined by the top-k lists. The Multi-GS method reported in [10] further proposes a different similarity measure between the top-k lists based on the sublist intersection. In [19], three operations, also called moves, are proposed to search the parameter space, namely, birth, death and local update, where a new structure hypothesis is created, deleted or the existing ones are updated, respectively. This three-moves strategy is embedded in a simulated annealing framework, where the previously mentioned Multi-GS approach [10] is used as a proxy to sample new hypotheses in the birth move.

All the above approaches create new hypothesis by sampling minimal subsets of points. Structures instantiated by minimal subsets of points are not very stable, especially when these points are very close to each other. In such cases, the resulting structures may not generalize well to other regions in the space and some true inliers may be incorrectly considered as outliers. Pham *et al*. [20] proposes to use the Random Cluster Model in order to create new hypothesis using subsets of points of arbitrary size. Following a similar idea as in [15], they enforce points belonging to the same structure to be close in the space.

Agglomerative correspondence clustering (ACC) [11] was presented to find multiple structures by clustering the set of correspondences using a pre-defined distance measurement. However, this method is restricted to the homography case.

We propose a novel approach that draws inspiration from some of the ideas of previous methods to provide a robust algorithm for multi-structure fitting. Similarly to [15, 20] we assume that points in the same structure are likely to be close in space, thus forming clusters. We also use inlier sets of arbitrary size to instantiate candidate structures, thus enhancing the stability of the results. As in [16], we seek the set of structures that best fits the data taking into account that they do not overlap, but contrarily to [16] we do not simply rank the existing candidate structures but actually select a sub-set of them. In contrast with all previous approaches, our method exploits the hierarchical relationships of the point-sets in order to find the structures representing the objects at the best scales.

## Method

Our main goal is to find multiple structures from two images represented by two point-sets. Following a coarse-to-fine strategy, we initially generate candidate structures using agglomerative clustering of the point-sets on each image, thus allowing to represent objects at different scales. Finding the multiple structures is then regarded as a problem of estimating the cluster-wise correspondences, each one representing a structure, so that the sum of inliers across structures is maximized and the selected clusters in the same image do not overlap. Maximizing the sum of inliers pushes the method to find the maximum number of structures possible. As we will show, the non-overlapping constraint leads to selection of clusters representing the multiple structures at the correct scales.

### Problem formulation

Consider two sets of points *X* and *Y* extracted from two different images of a scene with *n* and *m* points, respectively, and a set of putative correspondences *f* ⊂ {(1…*n*) × (1…*m*)}. A structure is a part of the scene (a subset of points in our case), showing a coherent motion between the two images according to some geometric transformation. For example, we can detect planar structures by seeking subsets of correspondences related by a homography, or we can detect rigid objects by seeking subsets of correspondences related by a fundamental matrix [8]. The subset of correspondences defining the structure are called the *inliers*, and the number of inliers within a correspondence-set *f*, denoted as *N*_*inl*_ (*f*), can be used as a quantitative measurement of the importance of the structure. A set of inliers for one specific geometric warp can be computed by methods like RANSAC [9].

In order to extend from the single- to the multiple-structure case, we follow a divide-and-conquer strategy where we first split the initial point-sets into candidate structures and then find the optimal candidates that maximize the sum of inliers across structures. To generate candidate structures we assume that points lying on the same structure are close to each other, thus forming clusters. We use agglomerative clustering to generate a set of candidate clusters in each image *P*_*i*_ ⊆ *X*, *i* = 1…*N*, and *Q*_*j*_ ⊆ *Y*, *j* = 1…*M* which are potentially able to represent structures of different sizes. Note that, in agglomerative clustering, clusters at larger scales are composed of the union of clusters at smaller scales.

**Fig 2:**
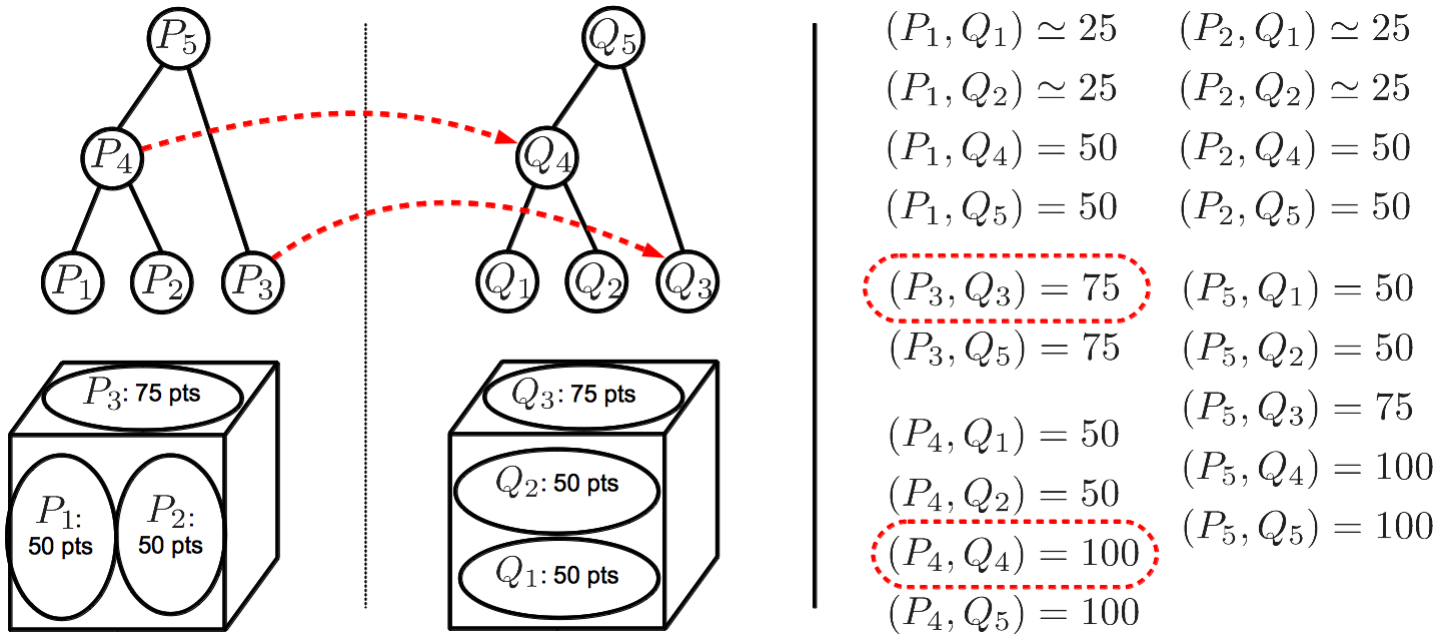
A toy example showing the process of finding the multiple structures (i.e., planar surfaces) in the two scenes of the cube shown in the left-hand side. The trees on top of each cube denote the structures generated by agglomerative clustering, where the leaf nodes are represented as circles on the cubes. On the right hand-side, we show the number of inliers found by RANSAC between each pair-wise cluster correspondence. The ground-truth solution that correctly matches the two surfaces is depicted with red dashed lines.

Finding multiple structures is then regarded as finding the subset of corresponding clusters *F* ⊂ {(1…*N*) × (1…*M*)} that maximize the sum of inliers across individual structures subject to the constraint that selected clusters in the same image cannot overlap. Enforcing clusters not to overlap means that we cannot select more than one cluster from the multi-scale representation covering a particular part of the image. This constraint, combined with the maximization of the number of inliers, drives the selection of clusters at the *best* scales, as we will see later. Fig 2 shows a toy example illustrating how the problem of multiple-structure discovery is related to the objective pursued by our method. The non-overlapping constraint in our method automatically discards all the solutions matching any pair of overlapping clusters in the same image (for example, selecting *P*_5_ or *Q*_5_ in Fig 2, would automatically discard all other clusters in their respective images). Given such a constraint, some of the top plausible configurations in decreasing order of inliers are the following: (1) {*P*_3_ → *Q*_3_,*P*_4_ → *Q*_4_} with 175 inliers, (2) {*P*_3_ → *Q*_3_,*P*_1_ → *Q*_2_,*P*_2_ → *Q*_1_} with 125 inliers, (3) {*P*_3_ → *Q*_3_,*P*_1_ → *Q*_1_,*P*_2_ → *Q*_2_} with 125 inliers, (4) {*P*_3_ → *Q*_3_,*P*_2_ → *Q*_4_} with 125 inliers, and so on. Note that the the objective pursued by our method (i.e., the plausible configuration with the highest number of inliers) corresponds to the ground-truth structures, denoted as red dashed lines in Fig 2.

Based on these definitions, we obtain multiple structures in the scene, each represented by a pair of corresponding clusters (*P*_*i*_,*Q*_*j*_), with (*i*, *j*) ∈ *F*, according to the following optimization problem:

$$\begin{array}{c} \arg\max_{F}\sum_{\left(i,j\right)\in F}N_{\text{inl}}\left(f_{ij}\right)\;\;\text{subject}\;\text{to}\\ P_{i}\cap P_{i^{\prime }}=⌀\;,\;Q_{j}\cap Q_{j^{\prime }}=⌀\;,\;\forall \;\left(i,j\right),\left(i^{\prime },j^{\prime }\right)\in F \end{array}$$ (1)

where *f*_*ij*_ ⊂ *f* is the subset of the initial correspondences between subsets *P*_*i*_ and *Q*_*j*_. This equation involves three main concepts: (1) each structure is represented by a pair of corresponding clusters (*P*_*i*_,*Q*_*j*_). By doing so, we can use standard methods such as RANSAC to evaluate each candidate structure; (2) we want to maximize the overall number of inliers across structures and (3) structures are constrained to be non-overlapping, thus being limited to select only a single cluster as representative of each structure among all the possible clusters at different scales covering that structure. This leads to the selection of the structures at the correct scales among all the possible candidates, as we will see later.

We will next show how to generate the candidate clusters *P*_*i*_ and *Q*_*j*_ representing the candidate structures.

### Generation of Candidate Point-Sets by Agglomerative Clustering

We assume that structures are located in spatially contiguous regions and, therefore, points belonging to the same structure are close to each other. Since structures can be of any size, we will need to generate point-sets at different scales. This is achieved by agglomerative clustering, which, given two point sets *X* and *Y* generates multi-scale representations of both images composed of point-sets *P*_*i*_, *i* = 1…*N*, and *Q*_*j*_, *j* = 1…*M*, representing structures of different sizes.

**Fig 3:**
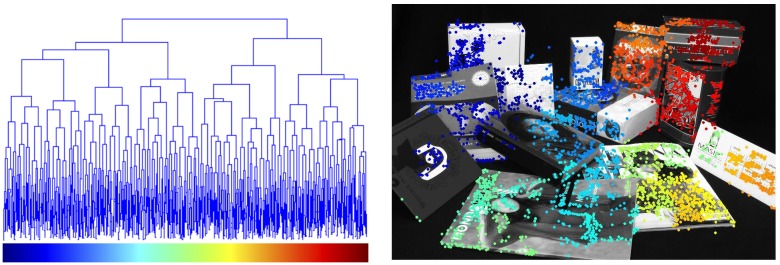
The left Figure shows the binary tree generated from agglomerative clustering of the point-set in the right Figure. Leafs (in the bottom of the tree) correspond to points in the image, each one being assigned a color (horizontal axis in the left Figure). Edges of the tree (blue lines) represent the cluster formations, where the higher clusters in the vertical axis are formed at later stages and convey higher-scale information (we refer the reader to the online version of the paper to appreciate the details).

At the beginning of agglomerative clustering, each point is regarded as a cluster. At each iteration, a new cluster is added resulting from the union of the two closest clusters. This process is repeated while there are at least two clusters that have not yet been involved in any union. Table 1 formalizes this clustering process, and Fig 3 shows an example of the results it provides.

**Table 1:** Agglomerative clustering.

**Input**: point-set *X*
**Output**: a pool of clusters in the image
**1**. Initialize a pool with clusters consisting of single points in *X*, and mark them as *unused*:
{*P*_*i*_ = {*x*_*i*_}, ∀*x*_*i*_ ∈ *X*}, Used_*i*_ = *false*
**2**. Find the two closest unused clusters in the pool:
argmin_*i*,*i*′_ *dist*(*P*_*i*_, *P*_*i*′_) s.t. Used_*i*_ = *false* ∧ Used_*i*′_ = *false*
**3**. Add a new cluster to the pool, denoted as *P*_*i*′′_, resulting from the union of the two closest clusters, i.e., *P*_*i*′′_ = *P*_*i*_ ∪ *P*_*i*′_
**4**. Mark *P*_*i*_ and *P*_*i*′_ as used and *P*_*i*′′_ as unused:
Used_*i*_ = *true*,Used_*i*′_ = *true*,Used_*i*′′_ = *false*
**4**. Repeat 2-4 until there is only one unused cluster

Clusters at the early stages are able to represent smaller structures whereas clusters at later stages are able to represent larger structures. As the result of the iterative union of clusters, some point-sets *P*_*i*_ shall be contained into other *P*_*i*′_ and, consequently, they shall have non-empty overlap; i.e, $P_{i}\cap P_{i^{\prime }}\neq ⌀$. The goal of our method is then to select the clusters at the appropriate levels representing the structures at the correct scales.

Rather than keeping the whole hierarchical structure, we discard the finest-scale information by only keeping the top *K* clusters in the binary tree closer to the root in breadth-first order. In our implementations we typically use *K* ≃ 60 which corresponds to approximately the 6 coarsest depth-levels of the binary tree. Note that by using such reduced trees, the finest-scale information available is the small clusters at the leaves. This follows a similar idea than the super-pixels approach in which the finer-level information (i.e., the pixels) is grouped into coarser representations [21]. In our case, the super-pixels correspond to the small clusters in the leaves of the reduced trees.

### Effects of the Non-Overlapping Constraint on the Selection of the Correct Scale

After having computed the multi-scale scene representations, we seek to maximize Eq (1) and estimate the subset of corresponding clusters, each one representing a structure, so that the sum of their inliers is maximized.

There exist two main issues that can prevent our method towards not selecting the best clusters: (i) selecting a cluster at a larger scale than a ground-truth structure when there are smaller clusters available; and (ii) selecting a cluster at a smaller scale than the ground-truth structure when there are larger clusters available. We will next show how the maximization of the number of inliers combined with the non-overlapping constraint tends lets us to handle both these issues and allows selecting the clusters representing the structures at the correct scales.

**Fig 4:**
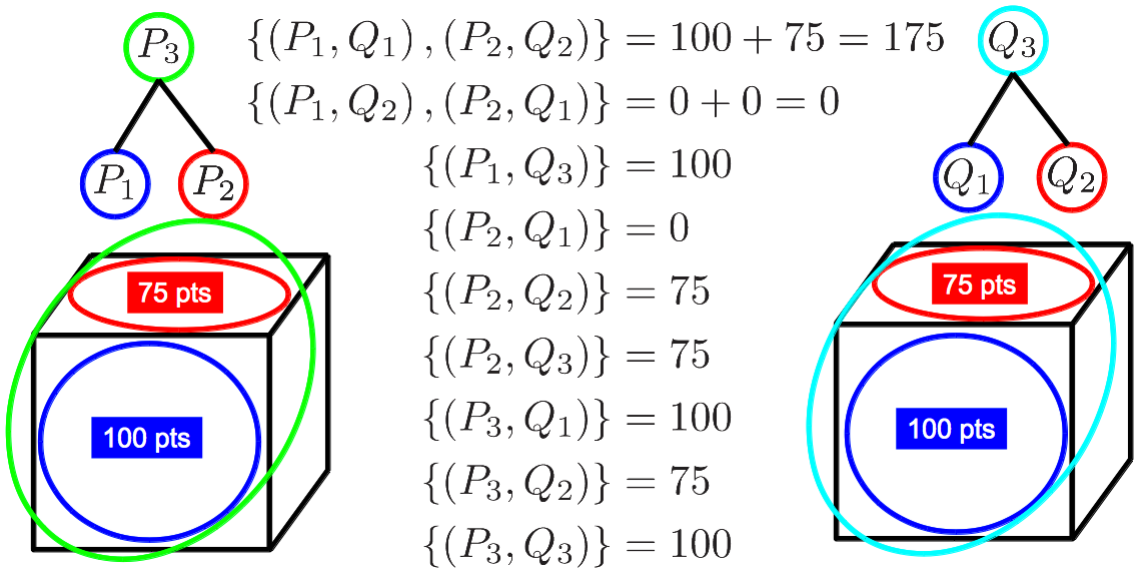
Penalty incurred when selecting clusters at a larger scale than the ground-truth structures. Each cube is a different image and each planar surface is a different structure. Ground-truth corresponding clusters are represented in the same color (along with their number of points). In the middle we show the topologically consistent configurations (i.e., matching non-overlapping clusters) along with the sum of inliers found by RANSAC for each configuration. The best configuration is the one matching the two structures at the correct scales (i.e., {(*P*_1_,*Q*_1_), (*P*_2_,*Q*_2_)}). Note that any configuration having into account the larger clusters (i.e., green and cyan circles) totally loses one of the structures (typically the smallest one) because, by definition, RANSAC is unable to simultaneously detect inliers in both structures.

Consider the case of selecting the pair (*P*_*i*_,*Q*_*j*_) made of two ground-truth structures instead of only one (i.e., selecting a structure at a larger scale than the correct one). When evaluating the local geometric consistency of candidate structure (*i*, *j*) using the function *N*_*inl*_ (*f*_*ij*_), points belonging to the smaller ground-truth structure will be lost, since RANSAC is only able to find the largest structure. The lost points cannot be recovered because, by to the non-overlapping constraint, no other subsets containing the missed points can be selected. This is illustrated in Fig 4.

**Fig 5:**
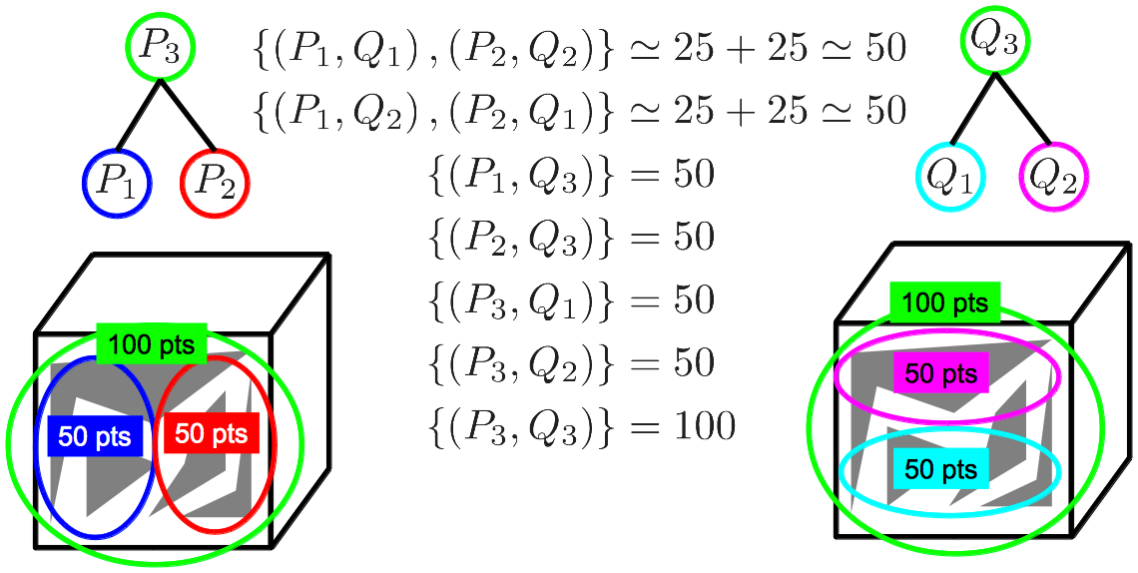
Penalty incurred by selecting smaller clusters than the ground-truth structures. Agglomerative trees (on top of each cube) have followed distinct aggregation paths due to the particular imaging conditions of each image. In the middle we show the 7 topologically consistent matching configurations (i.e., matching non-overlapping point-sets) along with the sum of inliers found by RANSAC in each case. We consider that sets *P*_1_ and *P*_2_ in the left image share approximately half of the ground-truth corresponding points with sets *Q*_1_ and *Q*_2_ in the right image (i.e., ≃ 25 points), respectively, because they only overlap partially. Among the 7 possible matching configurations, the best score is obtained by selecting the clusters at the correct scales.

On the other hand, consider the situation of choosing a pair of clusters (*P*_*i*_,*Q*_*j*_) at a smaller scale than the ground-truth structure. It is reasonable to assume that differences between the aggregation of points in the two scenes are more likely to happen at the smaller scales rather than at the larger scales. This means that, given a pair of clusters from different images *P*_*i*_ and *Q*_*j*_ lying on the same ground-truth structure, the smaller they are (i.e., from the earlier stages of the agglomerative clustering), the less likely they are to contain 100% of corresponding points. This implies that, given a single ground-truth structure, solutions *F* composed of many small-scale pairs will have lower chances of containing all the points in the structure than results *F* composed of only one (or a few) larger-scale pairs, due to the aggregation differences. The non-overlapping constraint implies that the missed points shall not be recovered. This situation is illustrated in Fig 5.

### Optimization by the Maximum Weighted Clique Problem

In the following, we show how the problem of finding multiple structures as defined in Eq (1) can be formulated as the Maximum Weighted Clique Problem (MWCP).

A *clique* is a subset of nodes in a graph such that they are completely connected [22]. In the literature, the MWCP has been applied to the matching of hierarchical structures where the optimal match is derived from the maximum weighted clique of their association graph [23, 24]. The association graph is a representation used for matching purposes where each node represents a putative correspondence and edges represent pair-wise compatibility between pairs of correspondences. The association graph can hold weights on the nodes indicating the quality of each correspondence. The maximum weighted clique of an association graph is then the subset of correspondences with higher quality such that all of them are pair-wise compatible.

**Fig 6:**
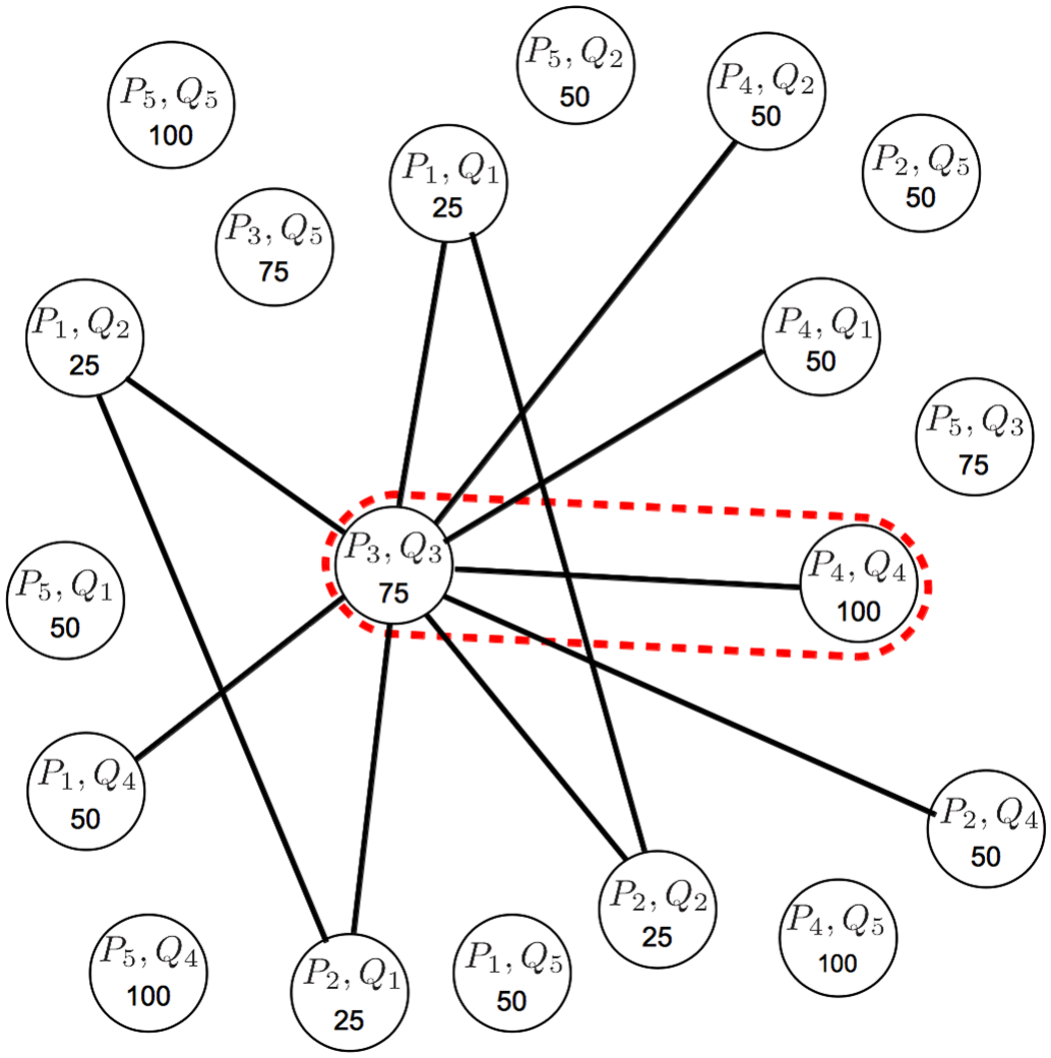
Association graph built from the problem in Fig 2. Each node represents a putative correspondence (i.e., a candidate structure) and each edge joins pairs of structures with non-overlapping clusters. The weight of each node is defined as the number of inliers (nodes with zero weight have been discarded). The maximum weighted clique (red dashed line), contains 2 nodes encompassing 175 inliers, which corresponds to the ground-truth structures (red dashed lines in Fig 2).

This has clear parallelism with our problem where we seek the subset of structures with maximum sum of inliers subject to the constraint that they are non-overlapping. In order to find multiple structures with the MWCP, we build an association graph where nodes are candidate structures consisting of all pair-wise correspondences between clusters, i.e., (*P*_*i*_,*Q*_*j*_), ∀ *i*, *j*. Since we want to maximize the sum of inliers across structures, we assign the weight of each node, denoted as (*P*_*i*_,*Q*_*j*_), according to the number of inliers of each structure, i.e., *N*_*inl*_ (*f*_*ij*_) (where *f*_*ij*_ ⊂ *f* is the subset of the initial correspondences between subsets *P*_*i*_ and *Q*_*j*_). We discard the nodes with null weights as they are not relevant. As defined by our problem, two structures will be compatible iff the pairs of involved clusters in their respective images do not overlap. Therefore, to constrain the solution of the MWCP to mutually compatible structures, we place edges between each pair of nodes, denoted as (*P*_*i*_,*Q*_*j*_) ∼ (*P*_*i*′_,*Q*_*j*′_), if and only if $P_{i}\cap P_{i^{\prime }}=⌀$ and $Q_{j}\cap Q_{j^{\prime }}=⌀$. Fig 6 shows an example of association graph.

### Implementation Details

Our method is general enough to use point-sets *X* and *Y* and correspondences *f* from any point-detection and correspondence-detection algorithms, respectively. We use SIFT [25] for both point and correspondence detection, since it is widely used for point matching [26–28]. We use the Euclidean distance between SIFT descriptors to decide the correspondences, although more sophisticated distance measures between histograms could also be used [29–31].

We use the graph degree linkage algorithm by Zhang *et al*. [32] to generate the agglomerative trees from the input images. This is an efficient graph-based agglomerative clustering algorithm that uses the product of the average indegree and average outdegree as a similarity measurement between clusters.

We use the model fitting algorithm RANSAC [9] to compute the number of inliers between the set of pairwise correspondences of Eq (1) to be used as weights for the nodes of the association graph. We will use different geometric models such as homography or fundamental matrix depending on the type of structures that we expect to find.

We use the implementation proposed in [33] for solving the MWCP which is based on a branch-and-bound algorithm with a better pruning strategy compared to similar counterparts.

## Results

In this section we present experiments and comparisons against state-of-the-art methods. We have performed experiments on different datasets that show the behavior of our method under a wide range of conditions. Additionally, we have created a dataset of our own, which we detail later. The main challenges of multi-structure robust fitting are retrieving as many structures as possible while minimizing the number of incorrect matches. We will therefore consider both these two variables to measure the performance of each method.

### Datasets

We present experiments on two datasets. The first dataset, called the *Book* multiple structure dataset, has been created by us and can be found at http://www.iri.upc.edu/research/webprojects/pau/datasets/msclique/ or osf.io/gbv7s. The second dataset is the widely used *Adelaide* multiple structure dataset [17], which is available at http://osf.io/gb5yc. We will perform homography estimation on the *Book* dataset and fundamental matrix estimation on the *Adelaide* dataset. For each dataset, wee will evaluate both the number of estimated structures and the number of incorrect matches not automatically discarded by the matching algorithms.

#### Book Dataset

The *Book* dataset has been created with the purpose of having a complex set of images containing much more structures than other available datasets and also showing different lighting conditions. The main purpose of the Book dataset is to test the homography estimation case and, therefore, structures consist of planar surfaces. By introducing light variations we will be able to assess the performance of all methods in a much wider range of conditions, as would happen in real case scenarios. Specifically, the variation of light conditions will have direct impact in the number of outliers, as features detected in one image may look radically different in other images, and hence may be not accurately detected, or not detected at all. Other datasets on which to perform multiple structure robust recognition usually introduce a very limited number of objects, and the algorithms evaluated on these scenarios typically saturate results, i.e, they perfectly retrieve all structures contained in the scene. This prevents from estimating the actual breaking point of the methods. The *Book* dataset is therefore intended to test not so favorable situations.

**Fig 7:**
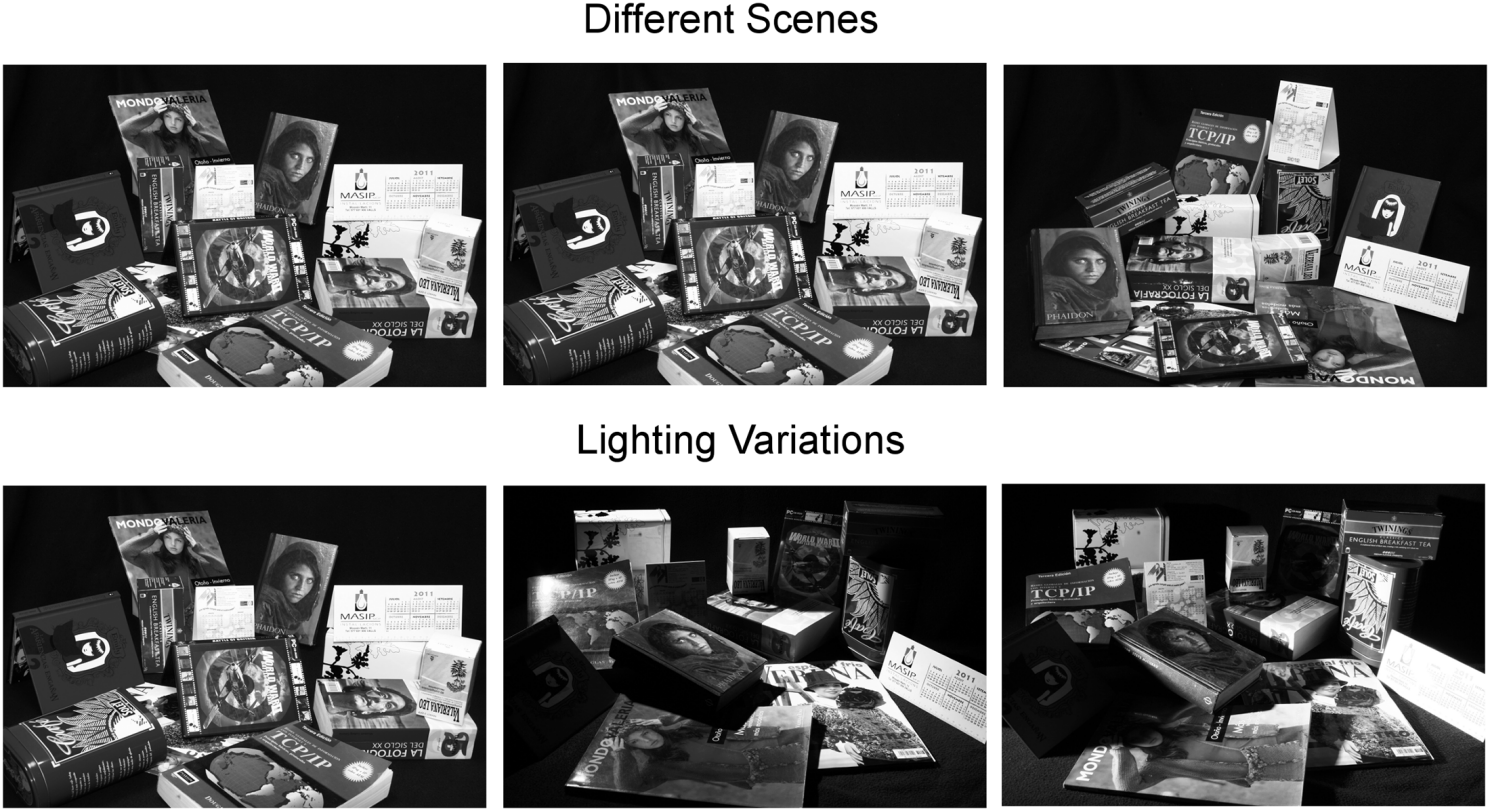
**First row:**Sample images from the 3 scenes in the *Book* dataset. **Second row:** Different illumination settings from one scene. The pairwise combination of the three different illumination settings between two scenes produces the 9 possible combinations.

We have introduced 3 different scenes, each scene containing up to 15 structures with 3 different illumination conditions (good illumination, mild shadows and severe shadows). When matching two scenes, we consider all possible combinations between any light condition, resulting in a total of 9 pairwise image matchings per each pair of scenes. Therefore, the performance of each method is assessed in a total of 27 matching experiments. Fig 7 shows the three scenes and the different lighting variations we have considered.

We used Lowe’s SIFT [25] to extract the point-sets *X* and *Y* from the two images to be matched. Tentative correspondences between each pair of points are determined based on the Euclidean distance between their SIFT descriptors, as done in [25]. We extract 4,000 interest points per image.

#### Adelaide Dataset

**Fig 8:**
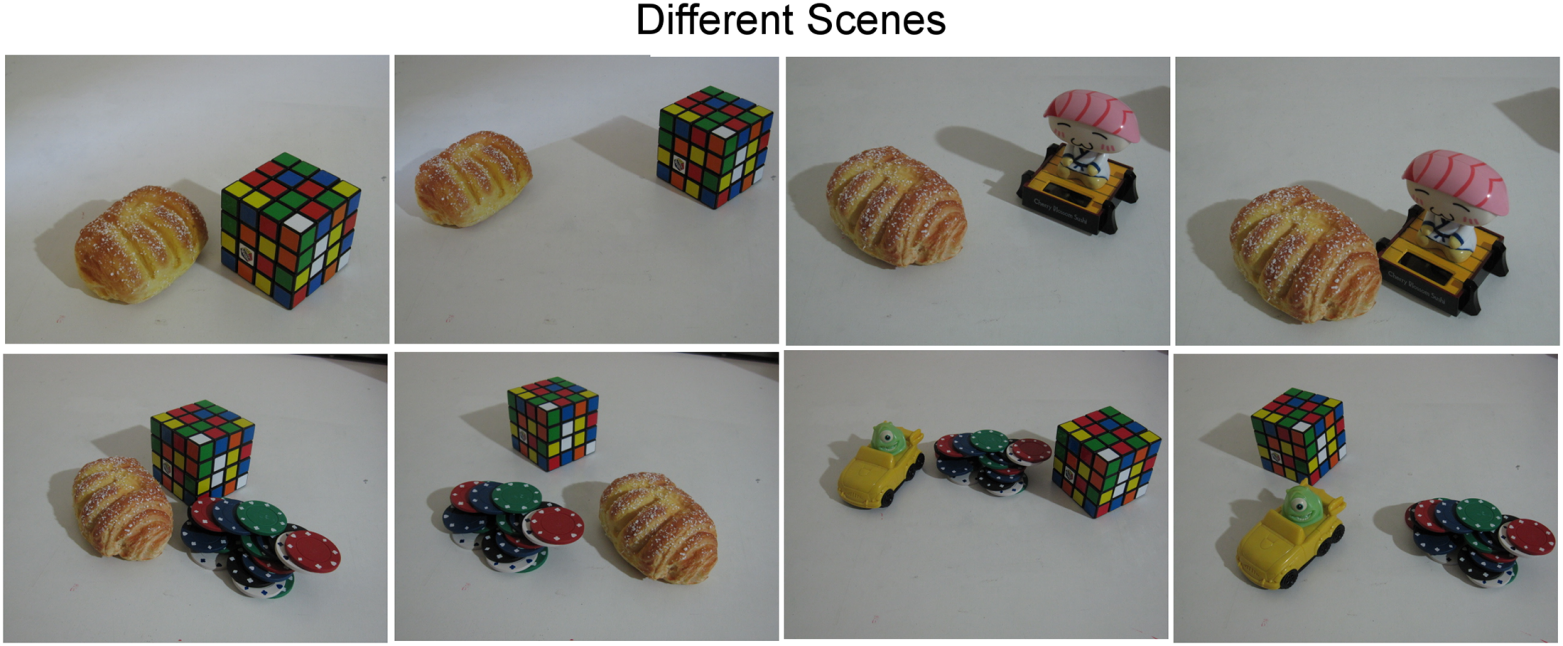
Sample images from the Adelaide dataset. Each image pair includes several objects with different motions that can be explained by a fundamental matrix. In this dataset there are no illumination changes that might affect the number of features detected on the same object between the two images.

The *Adelaide* dataset was presented in [17] (http://osf.io/gb5yc) and is the standard for testing robust multi-structure matching algorithms. Unlike in the Book dataset where we tested the homography estimation case, in the Adelaide dataset we intend to test the fundamental matrix estimation. By testing different fitting models across multiple datasets we can have further insights on the performance of all compared methods under different situations and image warps. The dataset contains several scenes showing either buildings or objects; the building scenes are used to evaluate a model based on homography estimation and only contain a single structure in terms of fundamental matrix estimation. Since we already assess the homography estimation performance in the *Book* dataset, we focus on the object scenes containing multiple structures for evaluating the fundamental matrix estimation performance. We show some sample example images of this dataset in Fig 8.

### Baselines

For Homography estimation the baseline algorithms we consider are the state-of-the-art methods Multi Guided Sampling (*MultiGS*) [10] and Agglomerative Correspondence Clustering (*ACC*) [11]. We compare homography estimation performance on both baselines. *MultiGS* by itself is not a robust matching algorithm but rather a sampling strategy. We perform a comparison using a pipeline similar to RANSAC but in which the sampling is not random but performed using Multi Guided Sampling. This sampling approach has showed to obtain state-of-the-art results when performing matching as can be seen in [10].

For Fundamental Matrix estimation the baseline algorithms we consider are: *MultiGS* [10], *PEARL* [15], *FLOSS* [14], *QP-MF* [16], *ARJMC* [19] and *RCMSA* [20]. We exclude *ACC* from the comparison in the Adelaide dataset since it is not designed for fundamental matrix estimation. We performed 2 different experiments, a first and more detailed one comparing our approach and *MultiGS* with the aim of giving insights on the performance of the approach. On the second one, we compared the rest of the methods following a standard validation strategy already used in previous works.

The correspondences between points are the same for all approaches. We use the MATLAB implementations provided by the authors for the MultiGS and ACC methods and we use the reported performances for the rest of the methods.

### Homography Estimation

**Fig 9:**
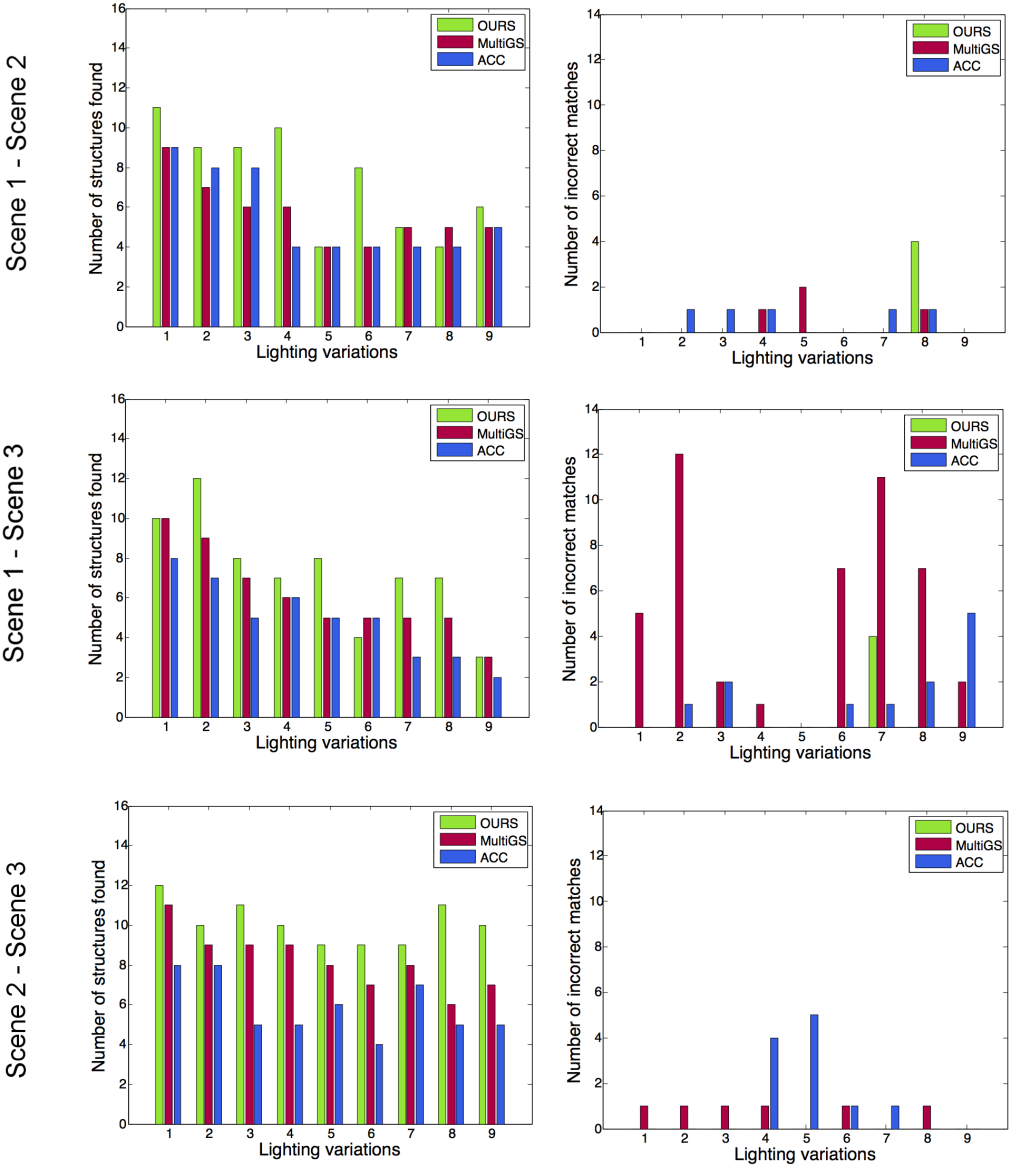
Homography estimation results on the book dataset. We show results for *MultiGS*, *ACC* and our method. Horizontal axis shows each particular experiment defined by a combination of lighting conditions between the two images to match. Vertical axis shows the number of correct structures detected (first column) and the number of incorrect matches produced (second column). As we can see, MSClique outperforms the competing approaches both in the number of correct structures and incorrect matches discarded.

The first model we tested is the homography estimation on the *Book* dataset. The inlier threshold for RANSAC has been set to 0.001, the time constraint has been set to 13 seconds and a maximum number of iterations to 25,000. We have tested each of the possible scene combinations and we show charts of the results in Fig 9, where each row accounts for a particular scene combination. Note how the proposed method consistently outperforms the other two baselines both in the number of correctly detected structures as in number of eliminated incorrect matches.

**Fig 10:**
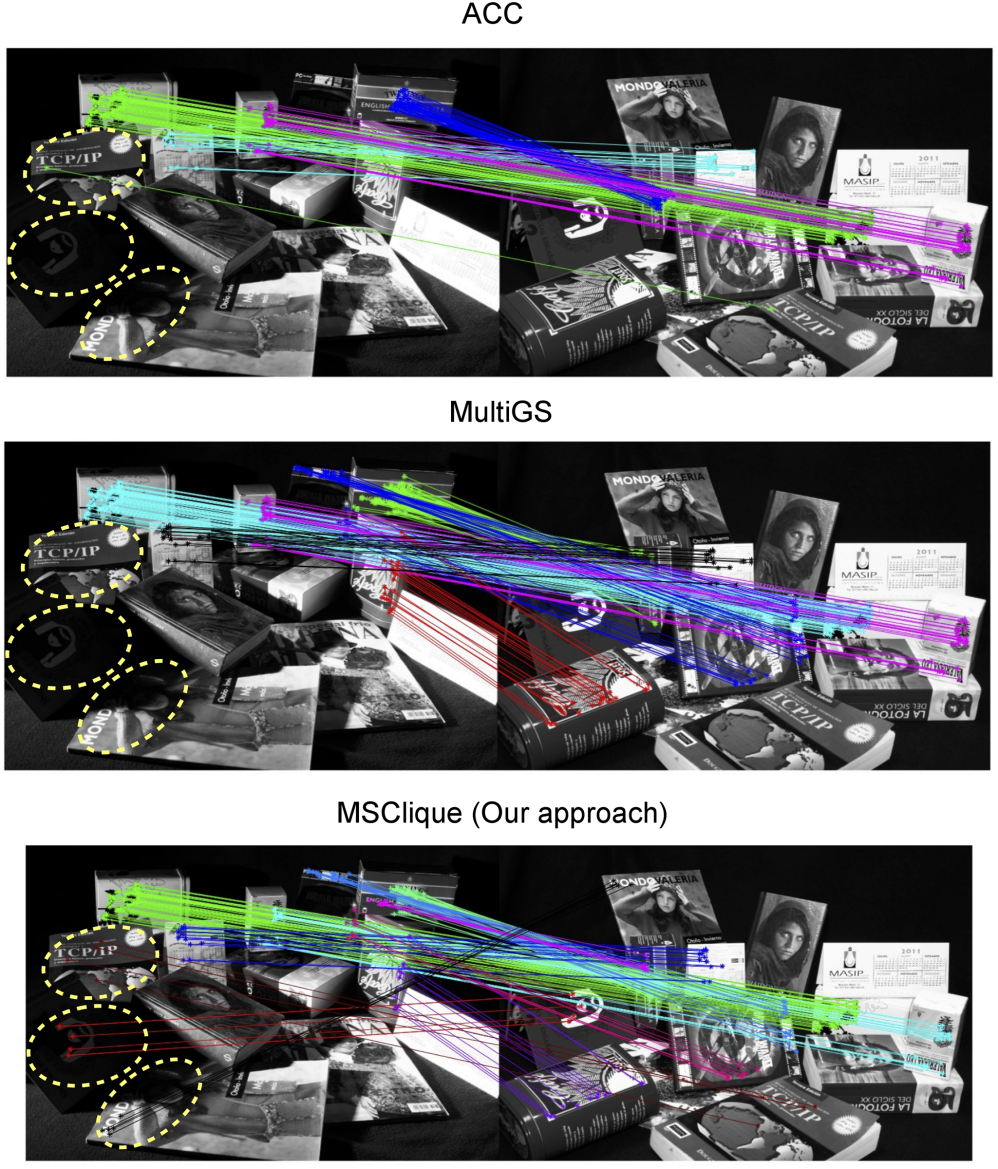
Examples results in the *Book* dataset. We obtain significantly more structures and produce less incorrect matches than any of the competing approaches. The yellow ellipses indicate difficult structures containing only a few matches that are found by our method and missed by the others.

As seen in Fig 10, the amount of change in illumination might significantly reduce the number of matches of a structure. This makes finding that structure much more difficult. If the number of inliers is small it becomes difficult to determine whether we have a correct structure or just incorrect matches that reproject close to the matching point.

As we have seen, MSClique outperforms state-of-the-art methods both in the number of structures found and in the number of incorrect matches correctly discarded. The proposed approach detects a higher number of structures than the rest of the methods because it groups points into clusters by taking into account their spatial relationships. This allows to even detect very small structures regardless the number of their inliers as long as they consistently match points in a cluster from one image to points in a cluster from the other image. This can be seen in the three structures in the left-hand side of Fig 10 highlighted with ellipses, which are only detected by our method.

### Fundamental Matrix Estimation

**Fig 11:**
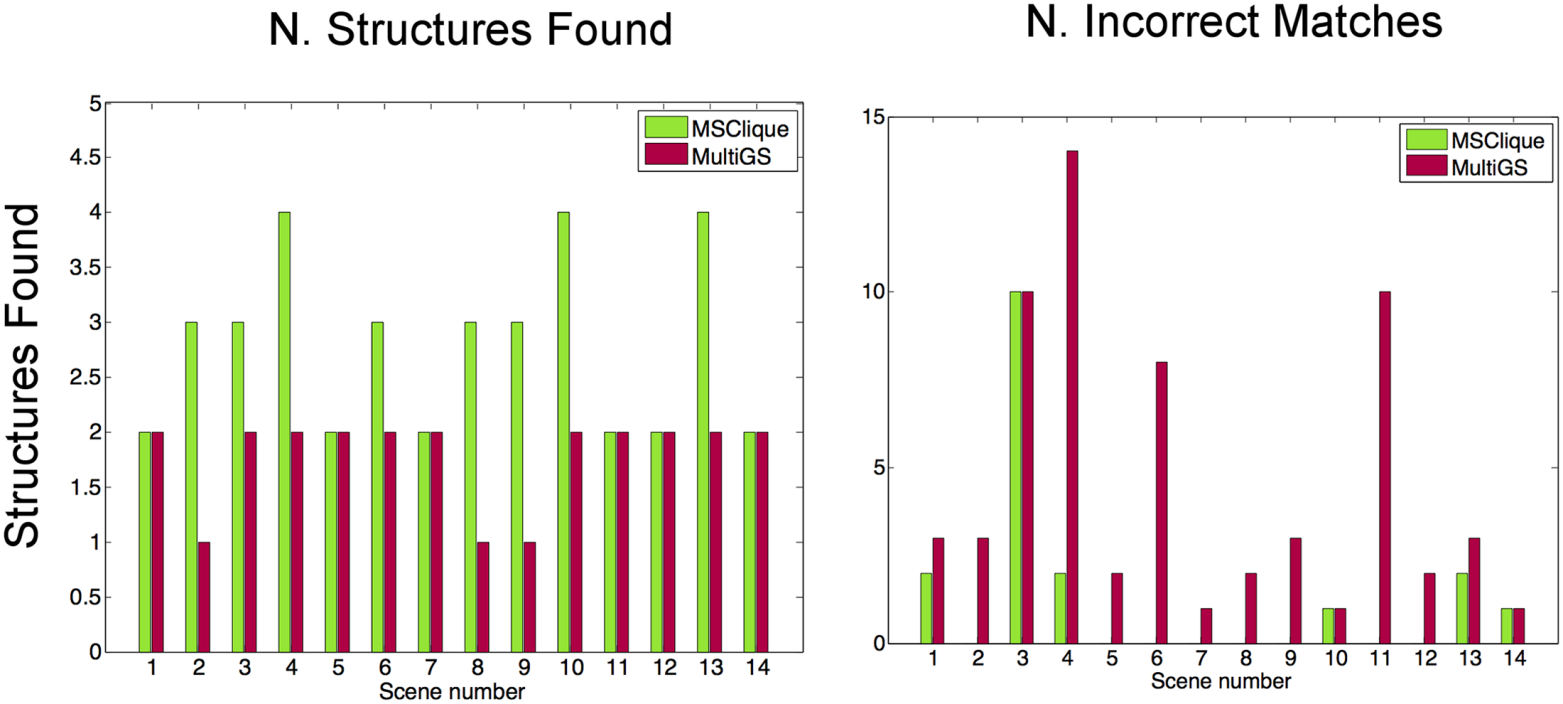
Fundamental matrix estimation results on the *Adelaide* dataset for *Multi GS* [10] and our approach. The first chart shows the number of structures found by each method. The second chart shows the number of incorrect matches produced by each method. If a structure contains incorrect matches we still count it as found. It can be seen that we obtain better results than the baseline method both in number of structures and number of incorrect matches discarded.

The second case we test is the fundamental matrix estimation on the *Adelaide* dataset [17] (http://osf.io/gb5yc). In this case, the inlier threshold has been set to 0.0001, the time constraint to 6 minutes and a maximum number of iterations is 25,000. The time constraint difference with respect to the *Book* dataset comes from the additional complexity required to estimate the fundamental matrix (estimating a fundamental matrix requires 8 point correspondences, while a planar homography can be estimated with 4 point matches). Fig 11 shows the results. Again, our approach yields better results than the baseline methods both in number of structures and in number of incorrect matches not eliminated.

We also compare the proposed method to the reported performance by a number of methods in a selected subset of challenging images from Adelaide dataset containing a high number of structures (we use the same images as in [20]). We use the following measure of segmentation error which penalizes both wrongful assignments and incorrect groupings of the data [20]:

$$\begin{array}{c} SE\left(g\right)=\min_{\Gamma }\frac{1}{C}\sum_{c=1}^{C}\delta \left(g_{c}^{\Gamma }\neq g_{c}^{⋆}\right)\;, \end{array}$$ (2)

where *g* is a vector assigning each initial correspondence in *f* a label denoting the estimated structure (with *C* being the total number of correspondences), *g*^⋆^ is the ground-truth structure membership variable and Γ denotes a permutation over the structure labels. Each structure in our method corresponds to each estimated cluster-wise correspondence (*i*, *j*) ∈ *F*. Therefore, all the correspondences contained in the same cluster-wise correspondence will be assigned the same structure identifier.

**Fig 12:**
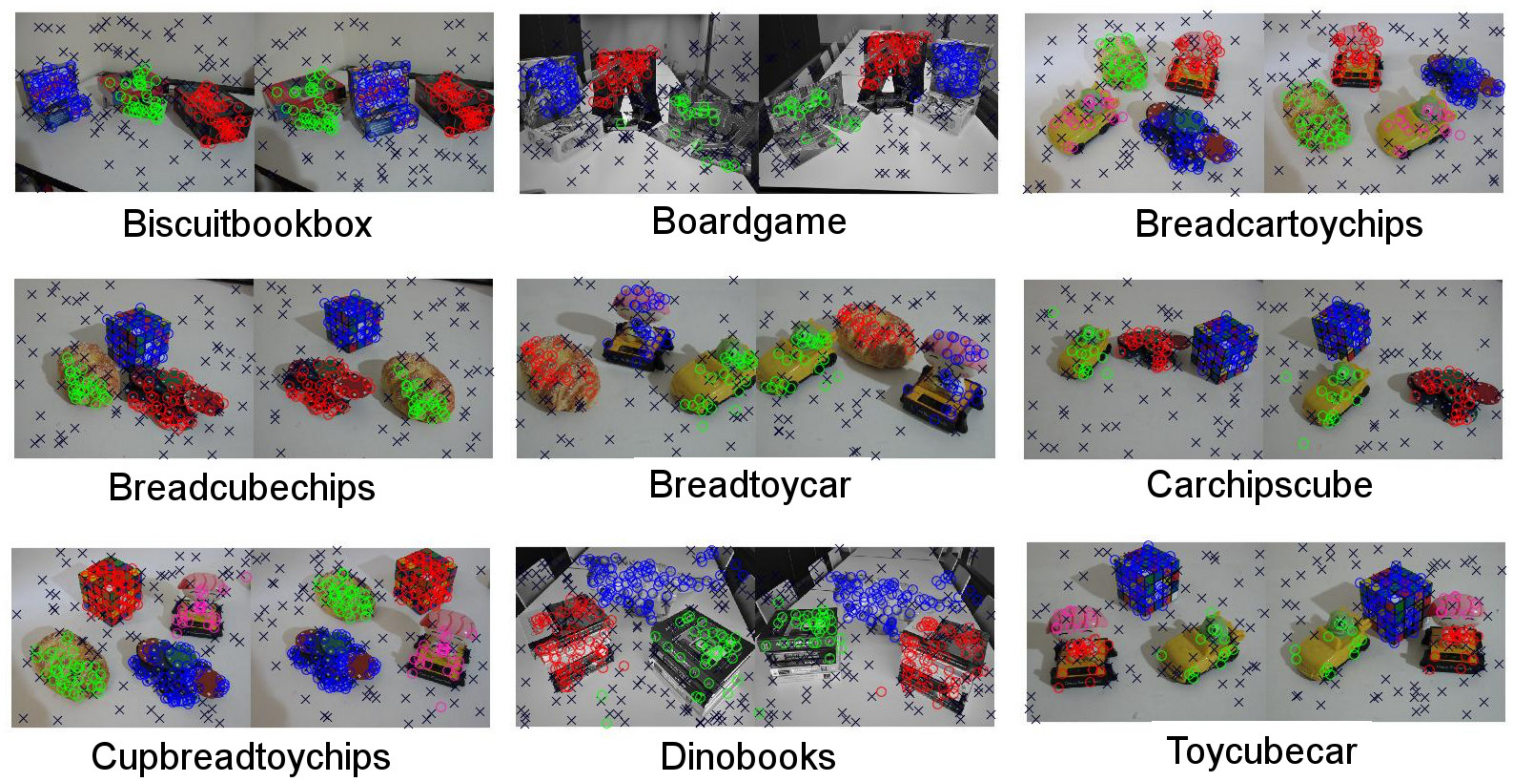
Qualitative results on the adelaide dataset. Points belonging to the same structure are denoted by same-color circles in both images. Outliers are denoted as black crosses.

Table 2 shows the quantitative performance of each method on each of the images and Fig 12 shows the qualitative results by our method.

**Table 2:** Quantitative segmentation results.

Datasets	Segmentation Error					
	PEARL	QP-MF	FLOSS	ARJMC	RCMSA	MSClique
Biscuitbookbox	8.11	7.72	11.58	11.58	7.72	**2.32**
Boardgame	16.85	17.20	17.92	19.35	12.90	**11.82**
Breadcartoychips	12.24	10.97	15.82	13.92	9.92	**8.43**
Breadcubechips	9.57	6.96	11.74	10.43	9.78	**6.95**
Breadtoycar	10.24	**8.73**	11.75	10.84	**8.73**	15.66
Carchipscube	10.30	9.09	16.97	15.76	**4.85**	9.09
Cubebreadtoychips	9.02	**7.34**	11.31	9.94	8.87	9.48
Dinobooks	19.17	17.78	20.28	20.56	17.50	**11.11**
Toycubecar	12.00	**10.50**	13.75	13.50	11.00	14.00

When considering the comparisons in Table 2, it can be clearly be seen that our approach outperforms the rest in most cases. As we can see, the average performances indicate that our approach outperforms the rest in 5 out of 9 objects, while the second best approach outperforms the rest in only 3. As shown in Fig 12, the segmentations of the different objects obtained by the proposed method are correct in most of the cases. Also, it is worth noting the significant advantage obtained by our method in the *Biscuitbookbox* and *Dinobooks* images. An analysis of the different characteristics of the methods suggests that the success of *QP-MF* might be due to the strategy of enforcing the non-overlapping constraint between pairs of structures. A similar conclusion could be drawn about the effectiveness of the strategies adopted by *RCMSA*, namely, (i) instantiating the structures using non-minimal subsets of points and, (ii) assuming that points in the same structure tend to be clustered in space. In light of this, the apparent success of the proposed method could be due to the fact of containing all these seemingly successful characteristics as well as exploiting the hierarchical relationships of the point-sets to drive the selection of structures at the correct scales.

## Discussion

There are two important factors influencing the performance of multiple-structure discovery (MSD) methods, namely, the accuracy of point-pattern matching and the particularities of the geometric arrangement of the objects in the scene. Therefore, it is important to consider such factors when evaluating robust MSD methods.

The performance of point-pattern matching is mainly affected by variations in the imaging conditions, such as lighting changes. Although advanced local feature matching methods such as SIFT [25] are to some extent robust to such conditions, they are still affected by severe changes such as drastic lighting variations as the ones shown in the *book* dataset. One of the aims for creating the *book* dataset was to evaluate the robustness of MSD methods under such conditions. As shown in Fig 10, the rate of detections in some structures can get reduced to just a couple of correspondences. Under such circumstances, methods adopting a random sampling strategy [10, 17, 18] are particularly prone to missing structures because, by definition, they have fewer chances of picking the smaller ones. Another limitation of the methods using the random sampling strategy, is that they instantiate the structure hypotheses using minimal subsets of points, which can decrease the reliability of the results, as already pointed out in [20]. Similarly as in [20], our method instantiates structure hypotheses using larger-than-minimal subsets of points. Moreover, our method incorporates the novel property of generating the structure hypotheses via agglomerative clustering of the point-sets, thus exploiting the geometric information at multiple scales. Results in Fig 9 demonstrate that this strategy is more robust under such conditions.

There is also the risk of under-segmenting the objects given some particularities in their geometric arrangement across the scene. For example, in the homography case, a co-planar arrangement of the surfaces of several objects may lead to under-segment them as a single object. Incorporating the prior knowledge that points in the same structure usually lie close to each other, helps preventing to wrongly merge objects which are far apart. This is the strategy followed by our method and also by [14, 20]. We argue that the success of our method in avoiding under-segmentation and correctly detecting a higher number of structures, as shown in Figs 11 and 12 and in Table 2, is due to the unique combination of the following features: (i) enforcing detected structures to be non-overlapping as in [16], (ii) instantiating hypothesis using non-minimal subsets of clustered points as in [14, 20], (iii) exploiting the hierarchical organization of the points.

The issues of under- and over-segmentation can be modelled in our method via the parameter *K*, namely, the number of clusters in the binary tree in breadth-first order. The larger the value of *K*, the deeper the trees and the smaller the clusters in the leaves. In fact, this value defines the size of the smallest object we are able to segment. If K is too small, some objects will already be under-segmented in the leaves. This parameter defines a trade-off between segmentation fineness and efficiency, since larger *K* values produce larger association graphs which require longer time to be solved for. In our case, a value of *K* = 60 produces good results in the order of seconds.

On the other hand, over-segmentation occurs when one object is detected as various objects and, as opposed to under-segmentation, it does not imply losing any structure. In our method, over-segmentation may occur when the aggregation of points produced when performing the agglomerative clustering follows exactly the same pattern in both images and the number of inliers by a single whole-structure match is the same as the sum of inliers in many smaller-scale matches. Such a case violates the assumption illustrated in Fig 5 and as result, our method has no longer preference for the whole-structure match. We have rarely encountered this issue with the proposed configuration. Although not desirable, this issue does not introduce any fitting error and can be easily solved in many contexts just by applying simple heuristics that prevent the transformations from the different clusters to be equivalent.

## Conclusions

Multi-structure robust matching is a complex task and most previous work tends to solve this problem using sampling strategies inspired on the RANSAC strategy, but adapted to the multi-structure case. RANSAC is effective in detecting the dominant structure in a scene, but it is not designed to simultaneously find multiple structures. Related approaches for multi-structure fitting acknowledge this fact and use different statistical techniques to guide the random sampling towards the discovery of multiple-structures. Nevertheless, sampling strategies have limited ability in finding the less dominant structures. We approach this problem in a fundamentally different way by exploiting the fact that points on the same structure are prone to be close to each other and then, we evaluate structures in terms of cluster-wise correspondences between images. With this procedure, we can even detect very small structures as long as there exist matches between corresponding clusters in the two images. As shown in the experiments, the proposed method detects a higher number of structures, while minimizing the number of incorrect matches, compared to other approaches.

## References

[1] Moreno-Noguer F., Lepetit V., Fua P., Pose priors for simultaneously solving alignment and correspondence, in: Computer Vision-ECCV 2008, Vol. 5303 of Lecture Notes in Computer Science, 2008, pp. 405–418.

[2] Serradell E., Ozuysal M., Lepetit V., Fua P., Moreno-Noguer F., Combining geometric and appearance priors for robust homography estimation, in: Computer Vision-ECCV 2010, Vol. 6313 of Lecture Notes in Computer Science, 2010, pp. 58–72.

[3] Penate-Sanchez A., Serradell E., Andrade-Cetto J., Moreno-Noguer F., Simultaneous pose, focal length and 2D-to-3D correspondences from noisy observations, in: British Machine Vision Conference, Bristol, UK, 2013, pp. 82.1–82.11.

[4] Sanromà G., Alquézar R., Serratosa F., A new graph matching method for point-set correspondence using the em algorithm and softassign, Computer Vision and Image Understanding 116 (2) (2012) 292–304. 10.1016/j.cviu.2011.10.009

[5] Sanromà G., Alquézar R., Serratosa F., Herrera B., Smooth point-set registration using neighboring constraints, Pattern Recognition Letters 33 (15) (2012) 2029–2037. 10.1016/j.patrec.2012.04.008

[6] Du S., Zheng N., Ying S., Liu J., Affine iterative closest point algorithm for point set registration, Pattern Recogn. Lett. 31 (9) (2010) 791–799.

[7] Sanromà G., Alquézar R., Serratosa F., Smooth simultaneous structural graph matching and point-set registration, in: Graph-Based Representations in Pattern Recognition, Springer Berlin Heidelberg, 2011, pp. 142–151.

[8] Hartley R., Zisserman A., Multiple View Geometry in Computer Vision, 2nd Edition, Cambridge University Press, New York, NY, USA, 2003.

[9] Fischler M. A., Bolles R. C., Random sample consensus: a paradigm for model fitting with applications to image analysis and automated cartography, Commun. ACM 24 (6) (1981) 381–395. 10.1145/358669.358692

[10] Chin T.-J., Yu J., Suter D., Accelerated hypothesis generation for multistructure data via preference analysis, IEEE Trans. Pattern Anal. Mach. Intell. 34 (4) (2012) 625–638. 10.1109/TPAMI.2011.169 21844630

[11] Cho M., Lee J., Lee K. M., Feature correspondence and deformable object matching via agglomerative correspondence clustering., in: ICCV, IEEE, 2009.

[12] Kanazawa Y., Kawakami H., Detection of planar regions with uncalibrated stereo using distribution of feature points, in: In British Machine Vision Conference, 2004, pp. 247–256.

[13] Zuliani M., Kenney C. S., Manjunath B. S., The multiRANSAC algorithm and its application to detect planar homographies, in: ICIP, 2005, pp. III–153.

[14] Lazic N., Givoni I., Frey B., Aarabi P., Floss: Facility location for subspace segmentation, in: Proceedings of the IEEE 12th International Conference on Computer Vision, ICCV’09, 2009.

[15] Isack H., Boykov Y., Energy-based geometric multi-model fitting, International Journal of Computer Vision 97 (2) (2012) 123–147. 10.1007/s11263-011-0474-7

[16] Yu J., Chin T. J., Suter D., A global optimization approach to robust multi-model fitting, in: Proceedings of the IEEE Conference on Computer Vision and Pattern Recognition, CVPR’11, 2011.

[17] Wong H. S., Chin T.-J., Yu J., Suter D., Dynamic and hierarchical multi-structure geometric model fitting, in: International Conference on Computer Vision (ICCV), 2011.

[18] Wong H. S., Chin T.-J., Yu J., Suter D., Mode seeking over permutations for rapid geometric model fitting, Pattern Recogn. 46 (1) (2013) 257–271. 10.1016/j.patcog.2012.07.005

[19] Pham T. T., Chin T. J., Yu J., Suter D., Simultaneous sampling and multi-structure fitting with adaptive reversible jump mcmc, in: Proceedings of the Conference on Advances in Neural Information Processing Systems, NIPS’11, 2011.

[20] Pham T. T., Chin T. J., Yu J., Suter D., The random cluster model for robust geometric fitting, IEEE Transactions on Pattern Analysis and Machine Intelligence 36 (8) (2014) 1658–1671. 10.1109/TPAMI.2013.2296310 26353345

[21] Ren X., Malik J., Learning a classification model for segmentation, in: Proceedings of the Ninth IEEE International Conference on Computer Vision—Volume 2, ICCV’03, 2003.

[22] Barrow H. G., Burstall R. M., Subgraph isomorphism, matching relational structures and maximal cliques., Inf. Process. Lett. 4 (4) (1976) 83–84. 10.1016/0020-0190(76)90049-1

[23] Torsello A., Hancock E. R., Computing approximate tree edit distance using relaxation labeling, Pattern Recogn. Lett. 24 (8) (2003) 1089–1097. 10.1016/S0167-8655(02)00255-6

[24] Pelillo M., Siddiqi K., Zucker S. W., Matching hierarchical structures using association graphs, IEEE Trans. Pattern Anal. Mach. Intell. 21 (11) (1999) 1105–1120. 10.1109/34.809105

[25] Lowe D. G., Distinctive image features from scale-invariant keypoints, Int. J. Comput. Vision 60 (2) (2004) 91–110. 10.1023/B:VISI.0000029664.99615.94

[26] Sanroma G., Alquézar R., Serratosa F., et al, Graph matching using sift descriptors-an application to pose recovery of a mobile robot, in: VISAPP, 2010.

[27] Sanromà G., Alquézar R., Serratosa F., Attributed graph matching for image-features association using sift descriptors, in: Structural, Syntactic, and Statistical Pattern Recognition, Springer Berlin Heidelberg, 2010, pp. 254–263.

[28] Sanromà G., Alquézar R., Serratosa F., A discrete labelling approach to attributed graph matching using sift features, in: Pattern Recognition (ICPR), 2010 20th International Conference on, IEEE, 2010, pp. 954–957.

[29] Serratosa F., Sanromà G., A fast approximation of the earth-movers distance between multidimensional histograms, International Journal of Pattern Recognition and Artificial Intelligence 22 (08) (2008) 1539–1558. 10.1142/S0218001408006880

[30] Serratosa F., Sanromà G., An efficient distance between multi-dimensional histograms for comparing images, in: Structural, Syntactic, and Statistical Pattern Recognition, Springer Berlin Heidelberg, 2006, pp. 412–421.

[31] Serratosa F., Sanromà G., Sanfeliu A., A new algorithm to compute the distance between multi-dimensional histograms, in: Progress in Pattern Recognition, Image Analysis and Applications, Springer Berlin Heidelberg, 2007, pp. 115–123.

[32] Zhang W., Wang X., Zhao D., Tang X., Graph degree linkage: Agglomerative clustering on a directed graph, in: ECCV (1), Vol. 7572 of Lecture Notes in Computer Science, Springer, 2012, pp. 428–441.

[33] Östergård P. R. J., A new algorithm for the maximum-weight clique problem, Nord. J. Comput. 8 (4) (2001) 424–436.

